# Anomalous Advection-Dispersion Equations within General Fractional-Order Derivatives: Models and Series Solutions

**DOI:** 10.3390/e20010078

**Published:** 2018-01-22

**Authors:** Xin Liang, Yu-Gui Yang, Feng Gao, Xiao-Jun Yang, Yi Xue

**Affiliations:** 1 State Key Laboratory for Geomechanics and Deep Underground Engineering, China University of Mining and Technology, Xuzhou 221116, China; 2 School of Mechanics and Civil Engineering, China University of Mining and Technology, Xuzhou 221116, China; 3 College of Mathematics and Systems Science, Shandong University of Science and Technology, Qingdao 266590, China; 4 Institute of Geotechnical Engineering, Xi’an University of Technology, Xi’an 710048, China

**Keywords:** anomalous advection-dispersion model, general Liouville–Caputo fractional-order derivative, series solution, Laplace transform

## Abstract

In this paper, an anomalous advection-dispersion model involving a new general Liouville–Caputo fractional-order derivative is addressed for the first time. The series solutions of the general fractional advection-dispersion equations are obtained with the aid of the Laplace transform. The results are given to demonstrate the efficiency of the proposed formulations to describe the anomalous advection dispersion processes.

## 1. Introduction

Fractional differential equations have been widely applied to describe the anomalous phenomena in multiple scientific fields, such as physical chemistry, environmental engineering, biology, etc. [1,2,3,4]. In general, the fractional advection-dispersion models with the Caputo fractional derivative [5] may match the real observation better than the classical advection-dispersion models [6,7], which were applied to describe the transport of chemical pollutants in shale gas exploitation [8]. For example, the time- [9] and space- [10] fractional advection-dispersion models have been verified to be able to capture some non-Fickian transport. The tempered advection-dispersion models, such as the promising models, can capture the scale-dependent dispersion (see [5]) and predict the truncated power-law breakthrough curves very nicely (see [11]). In fact, the spatial evolution of the conservative solute molecules with the complex distribution is closely related to the physical and chemical interactions between them and the porous media [12,13,14]. To describe the characteristics of the solute molecules, the entropy solution was used to discuss a class of the fractional degenerate advection-dispersion models in [15]. The fractional transient advection-dispersion model for the entropy density of a reactive plume was discussed in [16].

Recently, a great many new fractional calculus operators have been proposed in [17,18,19,20,21]. For example, the Prabhakar fractional operator via the three-parameter Mittag-Leffler function was suggested in [17]. More recently, a family of the general fractional calculus operators containing the Mittag-Leffler functions were presented in [18]. In particular, Giusti and Colombaro [17] presented how these general fractional calculus operators can reduce to the classical fractional calculus operators. The new general fractional calculus operators of Mittag-Leffler type had been employed to characterize the anomalous relaxation behaviors [19], linear viscoelastic system [20] and anomalous diffusion [21]. The new general Liouville–Caputo fractional derivative operator of Wiman type was considered to describe the anomalous relaxation behavior in [18]. However, the new general Liouville–Caputo fractional derivative operator of Wiman type has not been applied to model the advection-dispersion processes.

In view of the above, the principal objective of this paper is to explore the anomalous advection-dispersion equation describing the pollutant transport in shale gas exploitation within the general Liouville–Caputo fractional derivative operator of Wiman type.

The remainder of the present work is arranged as follows. In Section 2, brief reviews of the definitions of the Mittag-Leffler functions and generations and the new general Liouville–Caputo fractional operator of Wiman type are presented. In Section 3, the series solutions of an anomalous advection-dispersion models via the general Liouville–Caputo fractional derivative operator of Wiman type are obtained. Finally, the conclusions are summarized in Section 4.

## 2. Mittag-Leffler Function and a New General Liouville–Caputo Fractional Derivative of Wiman Type

In this section, we introduce the family of the Mittag-Leffler function and the new general Liouville–Caputo fractional derivative operator of Wiman type.

### 2.1. Mittag-Leffler Functions

In this subsection, the definitions of the Mittag-Leffler functions are called (see [1,2,17,18,20,21,22]).

Let C, R and N be the sets of complex numbers, real numbers and positive integers, respectively.

**Definition** **1.** *The Mittag-Leffler function with one parameter, proposed by Gösta Magnus Mittag-Leffler in 1903, is defined as (see [23]):*

(1) $$E_{\vartheta }\left(\varphi \right)=\sum_{\lambda =0}^{\infty }\frac{\varphi^{\lambda }}{\Gamma \left(\vartheta \lambda +1\right)}\;$$

*where φ,ϑ∈C,Reϑ>0,λ∈N and $\Gamma \left(\cdot \right)$ is the Gamma function.*

**Definition** **2.** *In 1905, Wiman extended the Mittag-Leffler function with two parameters, given as [24]:*

(2) $$E_{\vartheta ,\varpi }\left(\varphi \right)=\sum_{\lambda =0}^{\infty }\frac{\varphi^{\lambda }}{\Gamma \left(\vartheta \lambda +\varpi \right)}$$

*where φ,ϑ,ϖ∈C,Reϑ>0 and λ∈N.*

**Definition** **3.** *A further extension of (1) and (2) with three complex parameters, proposed by Prabhakar in 1971, is defined as [25]:*

(3) $$E_{\vartheta ,\varpi }^{\tau }\left(\varphi \right)=\sum_{\lambda =0}^{\infty }\frac{{\left(\tau \right)}_{\lambda }}{\Gamma \left(\vartheta \lambda +\varpi \right)}\frac{\varphi^{\lambda }}{\lambda !}$$

*where φ,ϑ,ϖ,τ∈C,Reϑ,τ>0,λ∈N, and the Pochhammer symbol is*

(4) $${\left(\tau \right)}_{\lambda }=\tau \left(\tau +1\right)\ldots \left(\tau +\lambda -1\right)=\left\{\begin{array}{c} 1,\;\;\;\;\;\;\;\;\;\;\lambda =0\\ \frac{\Gamma \left(\tau +\lambda \right)}{\Gamma \left(\tau \right)},\;\lambda \geq 1 \end{array}\right.$$

**Definition** **4.** *The Laplace transform of the function $f\left(t\right)$ is defined by [26]:*

(5) $$L\left(f\left(t\right)\right)=f\left(s\right)=\int_{0}^{\infty }f\left(t\right)e^{-st}dt$$

*where L is the Laplace transform operator.*

The Laplace transforms of the family of the Mittag-Leffler functions are listed in Table 1 (see [18,22,27]).

### 2.2. A New General Liouville–Caputo Fractional-Order Derivative of Wiman Type

**Definition** **5.** *Let ϑ,β,ϖ∈R,0<ϑ<1 and 0<β+ϖ<1. A new general Liouville–Caputo fractional-order derivative of Wiman type is defined as [18]:*

(6) $$\left({}_{0}^{LC}D_{t}^{\left(\vartheta \right)}\Omega \right)\left(t\right)=\int_{0}^{\gamma }{\left(\gamma -t\right)}^{\beta +\varpi -1}E_{\vartheta ,\beta +\varpi }\left({\left(\gamma -t\right)}^{\vartheta }\right)\Omega^{\left(1\right)}\left(t\right)dt$$

*where*

$$\Omega^{\left(1\right)}\left(t\right)=\frac{d\Omega \left(t\right)}{dt}$$

The Laplace transform of Equation (5) is given as [18]:

(7) $$L\left(\left({}_{0}^{LC}D_{t}^{\left(\vartheta \right)}\Omega \right)\left(t\right)\right)=s^{-\left(\beta +\varpi \right)}{\left(1-s^{-\vartheta }\right)}^{-1}\left(s\Omega \left(s\right)-\Omega \left(0\right)\right)$$

Remark.

If there exists [17]

$$\lim_{\eta \to 0}t^{\beta +\varpi -1}E_{\vartheta ,\beta +\varpi }\left(\eta t^{\vartheta }\right)=\frac{t^{\beta +\varpi -1}}{\Gamma \left(\beta +\varpi \right)}$$

then the Liouville–Caputo fractional derivative is the special case of the general Liouville–Caputo fractional-order derivative of Wiman type.

## 3. The Anomalous Advection-Dispersion Model with General Liouville–Caputo Fractional-Order Derivative of Wiman Type

### 3.1. The Model Background

**Figure 1:**
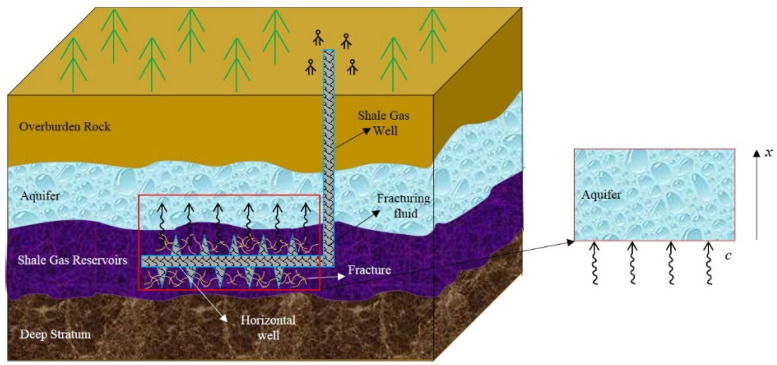
The mathematical model of the advection dispersion of the chemical pollutants in shale gas extraction involving general Liouville–Caputo fractional-order derivative of Wiman type.

The unconventional oil or gas (e.g., shale gas) development benefits many countries economically while bringing serious environmental pollution [8,28]. In this process, the fracturing fluids containing many chemical additives will diffuse into the aquifers through the porous media, resulting in the formation contamination [8]. The mathematical model of the advection dispersion of the chemical pollutants in shale gas extraction is shown in Figure 1.

The mathematical model of anomalous advection-dispersion process with general Liouville–Caputo fractional-order derivative of Wiman type is given by

(8) $$\left({}_{0}^{LC}D_{t}^{\left(\vartheta \right)}u\right)\left(x,t\right)=k\frac{\partial^{2}u\left(x,t\right)}{\partial x^{2}}-\rho \frac{\partial u\left(x,t\right)}{\partial x}\;\;\;\;x,\;t>0$$

with the initial value condition

(9) $$u\left(x,0\right)=0$$

and the boundary value conditions

(10) $$\left\{\begin{array}{c} u\left(0,t\right)=c\\ u\left(\infty ,t\right)=B \end{array}\right.$$

where $u\left(x,t\right)$ is the concentration of chemical pollutants in the aquifers, *k* is the dispersion coefficient of the aquifers, ρ is the seepage velocity of chemical pollutants, *c* is the concentration of chemical pollutants at the lower boundary of aquifers, and *B* is bounded.

### 3.2. The Series Solutions for General Fractional Advection-Dispersion Equation

Now, we find the series solutions for the general fractional advection-dispersion model within general Liouville–Caputo fractional-order derivative of Wiman type.

On performing the Laplace transform of Equation (8), we obtain

(11) $$s^{-\left(\beta +\varpi \right)}{\left(1-s^{-\vartheta }\right)}^{-1}\times \left(su\left(x,s\right)-u\left(x,0\right)\right)=k\frac{d^{2}u\left(x,s\right)}{dx^{2}}-\rho {\frac{du(x,s)}{dx}}_{}$$

where

$$L\left(u\left(x,t\right)\right)=u\left(x,s\right)$$

In a similar manner, the corresponding boundary-value conditions can be written as:

(12) $$\left\{\begin{array}{c} u\left(0,s\right)=\frac{c}{s}\\ u\left(\infty ,s\right)=B \end{array}\right.$$

Substituting Equation (9) into Equation (11), we have

(13) $$Mu\left(x,s\right)=k\frac{d^{2}u\left(x,s\right)}{dx^{2}}-\rho \frac{du(x,s)}{dx}$$

where $M=s^{1-\left(\beta +\varpi \right)}{\left(1-s^{-\vartheta }\right)}^{-1}.$

Making use of the eigenvalue method [8], we obtain the general solution of Equation (13), given as:

(14) $$u\left(x,s\right)=m_{1}e^{\frac{\rho +\sqrt{\rho^{2}+4kM}}{2k}x}+m_{2}e^{\frac{\rho -\sqrt{\rho^{2}+4kM}}{2k}x}$$

With the aid of Equation (12), we have

(15) $$u\left(x,s\right)=\frac{c}{s}e^{\frac{\rho -\sqrt{\rho^{2}+4kM}}{2k}x}=\frac{c}{s}e^{\frac{\rho }{2k}x}e^{\frac{-\sqrt{\rho^{2}+4kM}}{2k}x}$$

In order to obtain the solution of Equation (8) in the series form, we present

(16) u(x,s)=cseρ2kxe-ρ2+4kM2kx=cseρ2kx1-ρ2+4kM4k21122x+12ρ2+4kM4k2x2-16ρ2+4kM4k23322x3+124ρ2+4kM4k22x4+…=cseρ2kx+cseρ2kx∑n=1∞-xnknn22n!ΘnM

where

ΘnM=ρ24k+Mnn22

Furthermore, $\Theta_{n}\left(M\right)$ can be expanded as:

(17) ΘnM=ρ24k+Mnn22=ρ24knn22+n2ρ24knn22-1M+12!n2n2-1ρ24knn22-2M2+…+1N!n2n2-1…n2-N+1ρ24knn22-NMN+…=ρ24knn22+∑N=1∞n2n2-1…n2-N+1N!ρ24knn22-NMN

The substitution of Equation (17) into Equation (16) results in

(18) u(x,s)=cseρ-ρ2+4kM2kx=cseρ2kx+cseρ2kx∑n=1∞-xnknn22n!ρ24knn22+∑N=1∞AMN=cseρ2kx+ceρ2kx∑n=1∞-xnknn22n!s-1ρ24knn22+∑N=1∞As-1MN

where

A=n2n2-1…n2-N+1N!ρ24knn22-N

With the use of Table 1, we have

(19) $$L^{-1}\left(s^{-1}M^{N}\right)=t^{-N\left(1-\beta -\varpi \right)}E_{\vartheta ,1-N\left(1-\beta -\varpi \right)}^{N}{\left(t^{\vartheta }\right)}_{}$$

where $L^{-1}\left(\cdot \right)$ represents the inverse Laplace transform operator.

**Figure 2:**
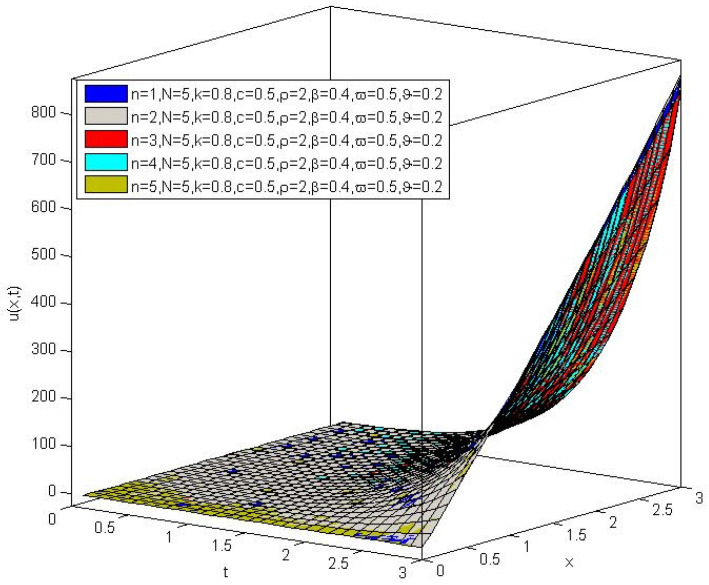
The concentration of chemical pollutants in the aquifers for the values n=1, n=2, n=3, n=4 and 5, and the parameters N=5, k=0.8, c=0.5, ρ=2, β=0.4, ϖ=0.5, ϑ=0.2.

Finally, substituting Equation (19) into Equation (18), we have the series solution of Equation (8) as:

(20) ux,t=ceρ2kx+ceρ2kx∑n=1∞-xnknn22n!ρ24knn22+∑N=1∞At-N1-β-ϖEϑ,1-N1-β-ϖNtϑ

and the corresponding plots of the changes of the concentration for the different parameters are displayed in Figure 2.

**Figure 3:**
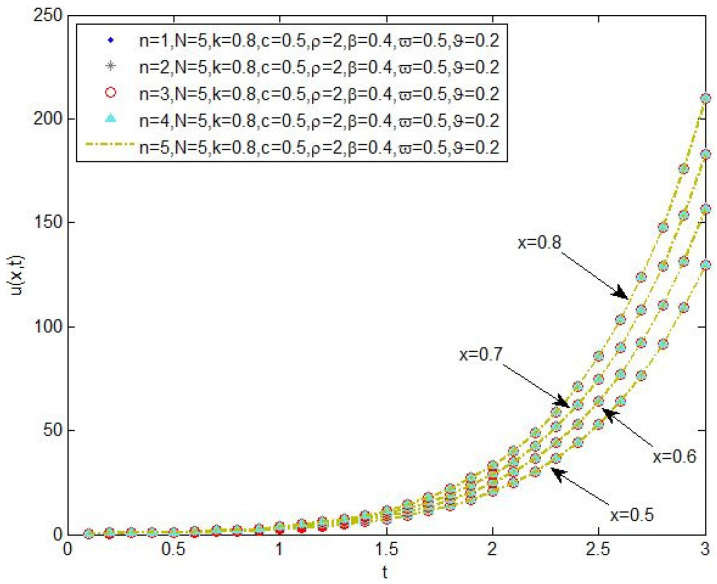
The changes of the concentration of chemical pollutants in the aquifers for the values n=1, n=2, n=3, n=4 and 5, and the parameters N=5,k=0.8,c=0.5,ρ=2,β=0.4,ϖ=0.5,ϑ=0.2 in the spaces x=0.5, x=0.6, x=0.7 and x=0.8.

The plot of the changes of the concentration of chemical pollutants in the aquifers for the values n=1, n=2, n=3, n=4 and 5, and the parameters N=5, k=0.8, c=0.5, ρ=2, β=0.4, ϖ=0.5, ϑ=0.2 in the spaces x=0.5, x=0.8, x=0.7 and x=0.8 is demonstrated in Figure 3.

**Figure 4:**
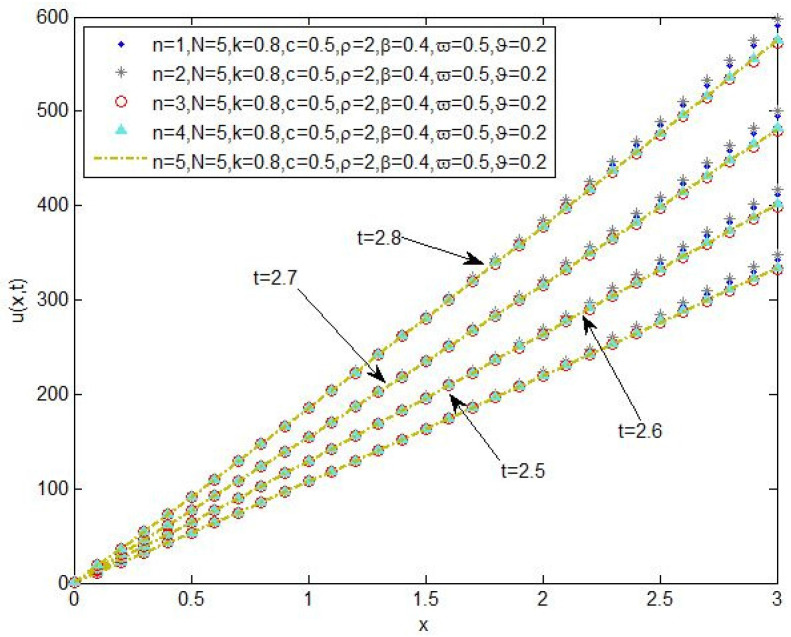
The change plot of the concentration of chemical pollutants in the aquifers for the values n=1, n=2, n=3, n=4 and 5, and the parameters N=5,k=0.8,c=0.5,ρ=2,β=0.4,ϖ=0.5,ϑ=0.2 in the times t=2.5, t=2.6, t=2.7, and t=2.8.

The plot of the changes of the concentration of chemical pollutants in the aquifers for the values n=1, n=2, n=3, n=4 and 5, and the parameters N=5,k=0.8,c=0.5,ρ=2,β=0.4,ϖ=0.5,ϑ=0.2 in the times t=2.5, t=2.6, t=2.7, and t=2.8 is shown in Figure 4.

## 4. Conclusions and Remarking Comments

In the current work, a novel anomalous advection-dispersion model with general Liouville–Caputo fractional-order derivative of Wiman type was proposed. The series solution of the anomalous advection-dispersion equation was obtained with the aid of the Laplace transform. The fractional advection-dispersion equation within fractional Liouville–Caputo fractional derivative is a special case due to the fact that the Liouville–Caputo fractional derivative is obtained by the general Liouville–Caputo fractional-order derivative of Wiman type. The proposed model is more efficient for the description of the anomalous advection-dispersion process than the classical model with Liouville–Caputo fractional-order derivative [9], which reduces to the classical advection-dispersion [6,7]. The results show that the general Liouville–Caputo fractional-order derivative of Wiman type is important for us to model power-law behaviors in nature.

## Figures and Tables

**Table 1:** The Laplace transforms of Mittag-Leffler functions with power-law functions.

Mittag-Leffler Functions with Power-Law Functions	Laplace Transforms
$t^{\beta -1}E_{\vartheta ,\beta }\left(t^{\vartheta }\right)\;\;\;\left(Re\vartheta ,\;\beta >0\right)$	$s^{-\beta }{\left(1-s^{-\vartheta }\right)}^{-1}$
$t^{\beta -1}E_{\vartheta ,\beta }^{\tau }\left(t^{\vartheta }\right)\;\;\;\left(Re\vartheta ,\;\beta ,\;\tau >0\right)$	$s^{-\beta }{\left(1-s^{-\vartheta }\right)}^{-\tau }$
$t^{\beta +\varpi -1}E_{\vartheta ,\beta +\varpi }\left(t^{\vartheta }\right)\;\;\;\left(Re\vartheta ,\;\beta ,\;\varpi >0\right)$	$s^{-\left(\beta +\varpi \right)}{\left(1-s^{-\vartheta }\right)}^{-1}$
$t^{\beta +\varpi -1}E_{\vartheta ,\beta +\varpi }^{\tau }\left(t^{\vartheta }\right)\;\;\;\left(Re\vartheta ,\;\beta ,\;\varpi ,\;\tau >0\right)$	$s^{-\left(\beta +\varpi \right)}{\left(1-s^{-\vartheta }\right)}^{-\tau }$
